# Heat stress compromises epithelial integrity in the coral, *Acropora hyacinthus*

**DOI:** 10.7717/peerj.6510

**Published:** 2019-02-26

**Authors:** Nikki Traylor-Knowles

**Affiliations:** Department of Marine Biology and Ecology, University of Miami, Rosenstiel School of Marine and Atmospheric Sciences, Miami, FL, United States of America

**Keywords:** Coral bleaching, Heat stress, Grainyhead, Wound healing, Thermal stress

## Abstract

It is well understood that heat stress causes bleaching in corals. Much work has focused on the way heat stress disrupts corals’ symbiotic relationship with endosymbiotic algal dinoflagellate, Symbiodiniaceae, a process called bleaching. However, the damage to the coral tissue that occurs during the bleaching process and, importantly, the factors that contribute to subsequent recovery, are not well understood. I hypothesize that the host tissue damage created by heat stress initiates cascades of wound healing factors that maintain epithelial integrity. These factors may be found to contribute to the coral’s potential capacity to recover. In this study, I present evidence that heat stress causes damage to the coral host tissue and that collagen is present in the gastrodermis of heat-stressed corals. I found that, during the early stages of bleaching, an important transcription factor for wound healing, Grainyhead, is expressed throughout the gastrodermis, where the cellular and tissue rearrangements occur. Lastly, using phylogenetics, I found that cnidarian Grainyhead proteins evolved three distinct groups and that evolution of this protein family likely happened within each taxonomic group. These findings have important implications for our study of coral resiliency in the face of climate change.

## Introduction

Corals (phylum: Cnidaria) are critical ecosystem builders that are important for promoting marine biodiversity, economic development, and human health ( Hughes et al., 2003). Reef building corals consists of polyps that secrete calcium carbonate, over which tissue and colonial polyps form. Within the endodermal epithelium, called the gastrodermis, many coral cells contain symbiotic dinoflagellates called *Symbiodinium* (recently renamed Symbiodiniaceae (LaJeunesse et al., 2018)). Through this critical partnership, the Symbiodiniaceae provides nutrients for the coral host, and in turn Symbiodiniaceae uses the wastes of the coral (Gates, Baghdasarian & Muscatine, 1992; Weis, 2008). During disturbance events such as heat stress, corals can “bleach”, disassociating from the Symbiodiniaceae partner, and the coral’s normally brown-pigmented tissue appears white as the skeleton shows through the translucent tissue. The phenomena of bleaching is highly variable, and different levels of bleaching can occur in different species of coral, as well as, under different conditions such as variable light intensities, salinity changes, and temperatures (Downs et al., 2009a; Downs et al., 2009b; Baker & Cunning, 2015; Brown & Dunne, 2015; Bieri et al., 2016). However, the signaling mechanisms leading to heat stress induced bleaching can occur very quickly, with the activation of stress response genes being upregulated within 150 min of heat stress (Traylor-Knowles et al., 2017). This indicates that the mechanisms for promoting bleaching are active well before the bleaching becomes visibly detectable (Traylor-Knowles et al., 2017). The cellular mechanisms that are activated include degradation of the symbiont within the coral host cell, coral host cell apoptosis, coral host cell necrosis, exocytosis of the symbiont from the host cell, and detachment of the host cell with the symbiont still within it (Gates, Baghdasarian & Muscatine, 1992; Weis, 2008). In this research article, I am defining a wound as a disruption of the epithelial integrity. I propose that the cellular damage of heat stress is, on a molecular level, akin to an epithelial wound, activating wound-healing pathways that could potentially help the coral recover from the damage.

In many previous gene and protein expression studies on heat stress in corals, factors known to be involved in wound healing have been identified, including many different collagens (Desalvo et al., 2008; Moya et al., 2012; Barshis et al., 2013; Bay et al., 2013; Kenkel et al., 2013; Maor-Landaw et al., 2014; Seneca & Palumbi, 2015; Rose, Seneca & Palumbi, 2015; Ricaurte et al., 2016). Collagen production is a hallmark of wound healing, and, in many organisms, is typically increased at the site of a wound during the late wound-healing phase (Diegelmann & Evans, 2004; Deonarine et al., 2007). In reaction to heat stress, both increases and decreases in gene expression of collagen were found (Table 1). For example, in the coral *Montastraea faveolata*, expression of collagen precursors was downregulated in response to heat stress, but in the coral *Acropora hyacinthus*, collagen expression was upregulated (Barshis et al., 2013; Desalvo et al., 2008). This variation in expression could be due to the different species of corals, the different types of collagens (there are more than 30 in the *Acropora digitifera* genome alone), and the different types of heat stress exposures that were performed in each study. Despite this variation, it is evident from these previous studies that collagens are reacting to heat stress in corals. Based on this observation, I hypothesize that heat stress creates damage to the host tissue, which in turn initiates cascades of wound healing factors that maintain epithelial integrity in response to the heat damage. One such cascade involves the Grainyhead transcription factor pathway.

**Table 1:** Collagen gene and protein expression patterns from previous coral and anemone heat stress studies.

**Collagen type**	**Experiment**	**Organism**	**Citation**
↓ procollagen type I, alpha 2	Heat stress and bleaching, microarray	*Montastraea faveolata*	Desalvo et al. (2008)
↑ collagen type IV	Chronic heat stress, qPCR transcriptome	*Porites astreoides*	Kenkel et al. (2013)
↑ collagen alpha-1(I) chain, ↑ mini collagens	Lab heat stress, transcriptome	*Acropora hyacinthus*	Barshis et al. (2013)
↓ collagen	Heat stress, microarray	*Stylophora pistillata*	Maor-Landaw et al. (2014)
↓ collagen	Heat stress, transcriptome	*Acropora hyacinthus*	Seneca & Palumbi (2015)
↑ collagen	Temperature acclimation, qPCR	*Acropora millepora*	Bay et al. (2013)
↓ collagen	Bleached versus unbleached, proteomics	*Acropora palmata*	Ricaurte et al. (2016)
↑ collagen	Transcriptional module heavily weighted for collagens, negatively correlated with bleaching outcomes	*Acropora hyacinthus*	Rose, Seneca & Palumbi (2015)
↓ Collagen alpha-1, ↓ Collagen alpha-2, ↓ Collagen-like	Heat and UV stress, microarray	*Anemonia viridis*	Moya et al. (2012)

Grainyhead (GRH) is a transcription factor which functions as a critical regulator of genes, including transglutaminase, dopa decarboxylase, and others that are crucial for tissue remodeling as has been shown in studies involving mice, *Xenopus*, and *Drosophila* (Mace, Pearson & McGinnis, 2005; Harden, 2005; Ting et al., 2005). In mice, GRH is involved in the development and maintenance of epithelial integrity (Harden, 2005; Ting et al., 2005). The mouse *GRH* is required during embryogenesis, where it is expressed exclusively in the developing ectodermal epithelium (Ting et al., 2005). Additionally, mouse GRH-like 2 is necessary for the expression of important adheren and tight junction genes in many different types of epithelia including the surface ectoderm and gut endoderm (Werth et al., 2010). Likewise, in *Xenopus*, *XGRH1* has been implicated in the development of the epidermis (Tao et al., 2005). In morpholino studies, knockdown of *XGRH1* led to loss of surface structures and pigmentation as well as neck and eye defects associated with epidermal instability (Tao et al., 2005). In *Drosophila,* GRH maintains the tension of the cuticle and induces cuticle development and repair following injury (Mace, Pearson & McGinnis, 2005; Moussian & Uv, 2005). In cnidarians, GRH has been was bioinformatically characterized in *Nematostella vectensis* (Traylor-Knowles et al., 2010). However, little is understood about the phylogenetic relationship of GRH among cnidarians, and even less is understood about its function.

In this study, I conducted a series of experiments to determine whether heat stress causes an epithelial disruption similar to a wound, thus activating wound-healing pathways. To test if coral epithelial integrity was compromised during a short-term heat stress, I used histology to identify cellular architectural changes. I then used *in situ* hybridization to examine spatial expression of *GRH* and found that *GRH* is expressed in the gastrodermis of the coral tissue, the same epithelium where bleaching occurs. I also examined the phylogenetics of cnidarian GRH and discovered that within the cnidarians tested, GRH had three distinct clades that are primarily driven by taxonomic grouping. This diversity of GRH within cnidarians could indicate a wider repertoire of functional significance.

## Materials and Methods

### Sample preparation

The samples examined in this study were part of three previously published genomic studies on coral heat tolerance in Ofu, American Samoa (Seneca & Palumbi, 2015; Rose, Seneca & Palumbi, 2015). These corals were originally from two separate pools: the highly variable (HV) pool and the moderately variable (MV) pool. These pools have been extensively studied due to their large temperature fluctuations, where corals in the HV pool can see regular temperatures over 32 °C and up to 35 °C at the hottest points during summer days (Craig, Birkeland & Belliveau, 2001; Oliver & Palumbi, 2009). Over a two day sampling period, small 50 mm long branches from six colonies of *Acropora hyacinthus* were collected at approximately 9 AM in the morning, and were placed into experimental tanks that were built to perform tightly regulated heat stress or control conditions (Barshis et al., 2013;  Palumbi et al., 2014; Seneca & Palumbi, 2015). Heat stressed corals were exposed to a four hour heat ramp from 29 °C to 35 °C, held for 1 h at 35 °C, and quickly returned to 29 °C (Seneca & Palumbi, 2015; Rose, Seneca & Palumbi, 2015; Traylor-Knowles et al., 2017). This heat stress profile was designed to mimic daily heat fluctuations measured in the HV pool during summer months. Controls were held at 29 °C for the duration of the experiment in temperature controlled tanks (Seneca & Palumbi, 2015; Rose, Seneca & Palumbi, 2015; Traylor-Knowles et al., 2017) Samples were visually assessed for bleaching, and assigned a score of 1 for no bleaching up to 5 for fully bleached (Fig. S1, Table S1) (Seneca & Palumbi, 2015; Rose, Seneca & Palumbi, 2015).

### Histological preparation

Ten heat-stressed branches, and two control branches from six colonies were preserved in 4% paraformaldehyde in filtered sea water, and washed in phosphate-buffered saline (PBS), and stored in methanol at −20 °C for later processing ( Wolenski et al., 2013) (Colony information in Table S1). RNAase-free conditions were used to prepare thin paraffin sections of coral branches. This was performed by IDEXX Laboratories Inc. (Columbus, MO, USA). Decalcification of the coral branches was done using Morse’s solution (25% formic acid, 10% sodium citrate). Paraffin infiltration was then performed, and tissue was embedded into paraffin. Before sectioning of the paraffin tissue blocks, the microtomy equipment was cleaned and treated for RNases using RNase Zap (Ambion, Inc. Houston, TX, USA) and DEPC-treated water (Sigma, St. Louis, MO, USA) was used in the water bath. For each samples, a new microtomy knife was used to prepare 5 µm sections. Using charged microscope slides (Leica Biosystems, Inc. Buffalo Grove, IL, USA), tissue sections were then mounted. For each sample, one section was processed for hematoxylin and eosin staining (H & E) ( Fischer et al., 2008). Additionally, 2–3 sections were processed for connective tissue and collagen staining using Masson’s trichrome stain (Goldner, 1938).

### *In situ* hybridization probe preparation

The details of this protocol were previously published in Traylor-Knowles et al. (2017). In short, the Digoxigenin (DIG)-labeled anti-sense, single-stranded mRNA probes were designed for *GRH*. Primers were designed to replicate amplicons approximately 400 bps in length for the *GRH* gene (Table S3).

### *In situ* hybridization

The details of this protocol were previously published in Traylor-Knowles et al. (2017) and (Traylor-Knowles, 2018). *In situ* hybridization was performed on unstained coral-tissue sections that were embedded in paraffin (Traylor-Knowles et al., 2017; Traylor-Knowles, 2018).

### Phylogenetic analysis

Sequences were collected from different genomic database resources (Table S2) by using BLASTp with a human GRH homolog. To determine the evolutionary history of GRH within cnidarians, the Neighbor-Joining method was employed using MEGA7 software (Kumar, Stecher & Tamura, 2016). Analysis was done on 22 protein sequences. Two thousand replicates of the bootstrap test were used to determine the percentage of replicate trees in which the associated taxa clustered together (Felsenstein, 1985). The tree was drawn to scale, with branch lengths in the same units as those of the evolutionary distances used to infer the phylogenetic tree. The evolutionary distances were computed using the Poisson correction method (Zuckerkandl & Pauling, 1965) and are in the units of the number of amino acid substitutions per site. All positions with less than 95% site coverage were removed. A total of 44 amino acids were analyzed after gaps were removed.

## Results and Discussion

### Coral tissue and cell integrity are damaged by heat

**Figure 1:**
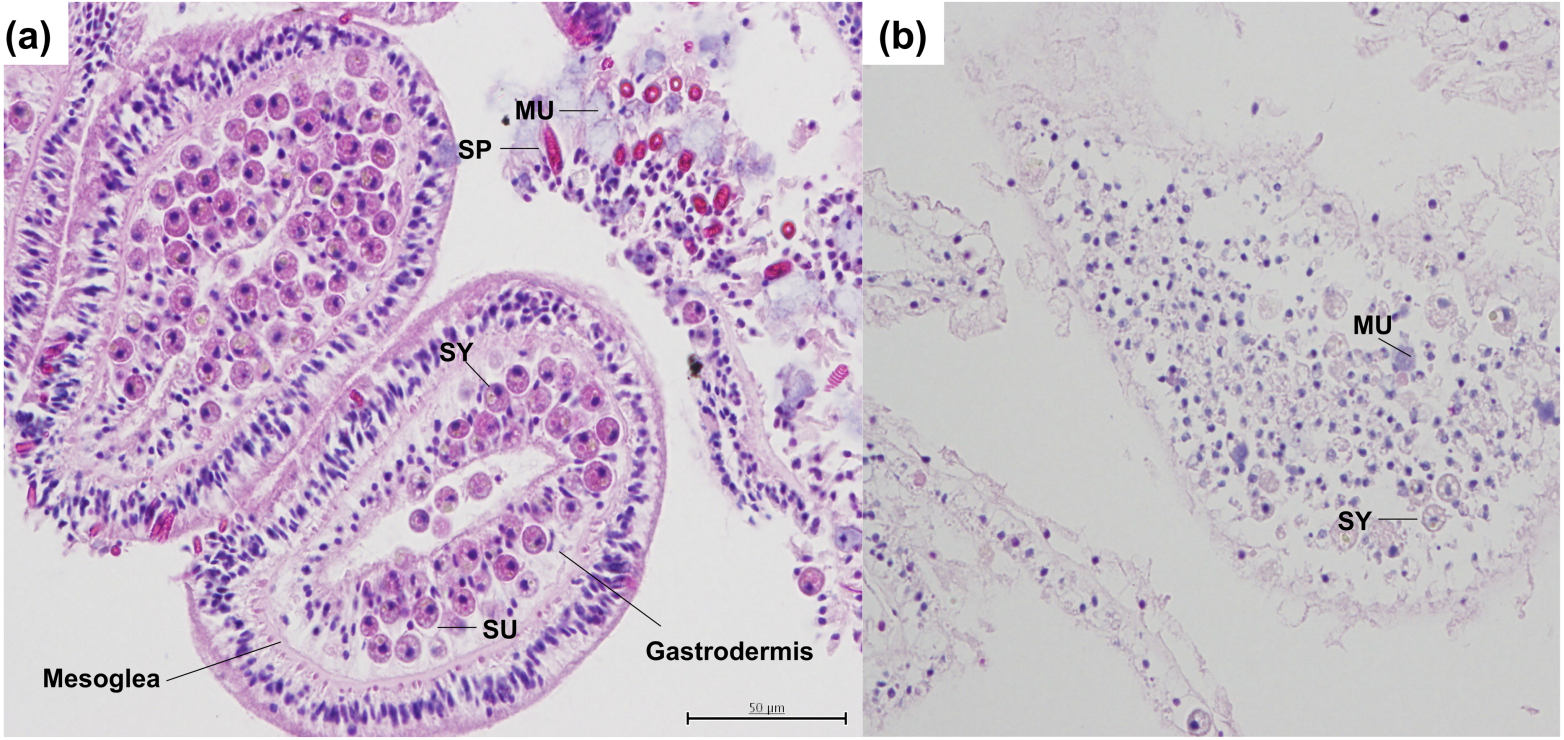
Histological serial cross sections of heat-stressed and control coral tissues, stained with H & E in the coral *A. hyacinthus*. (A) Sagittal cross section through control coral tissue, not exposed to heat-stress conditions. In the controls, tissue epithelia and mesoglea are intact with normal architecture and strong staining. *Symbiodiniaceae* are present within coral gastrodermal cells, and appear to be healthy and undamaged. Spirocytes are present and are not discharged or released from the epidermis. (B) Sagittal cross section through heat-stressed coral tissue. In the heat-stressed samples, epithelia and mesoglea are damaged. Few *Symbiodiniaceae* are present, with the exception of some that are necrotic. Swollen mucocytes are also present. Tissue staining is not as strong as compared with the control, indicating loss of proteins, vacuolation, and pycnosis of nuclei. Abbreviations: SY, *Symbiodiniaceae*; SP, Spirocyte; SU, symbiont containing gastrodermal cell; MU, mucocytes.

**Figure 2:**
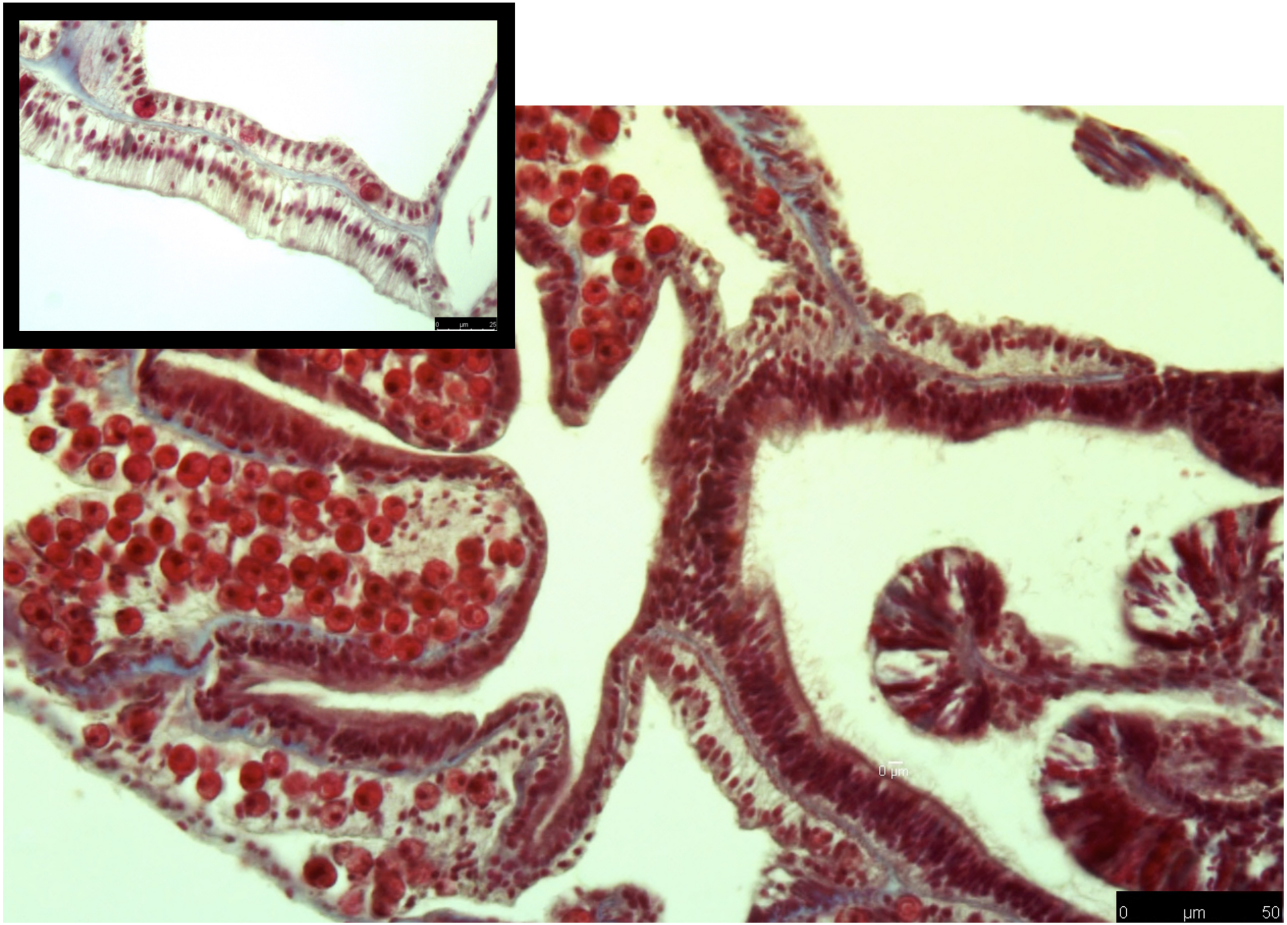
Masson’s trichrome-stained sections for collagen expression in control coral samples. Control samples along the edge of a gastrovascular cavity shows intact tissue layers, and clear staining. Collagen (blue) is present within the mesoglea, and intact *Symbiodiniaceae* are present in gastrodermal cells.

**Figure 3:**
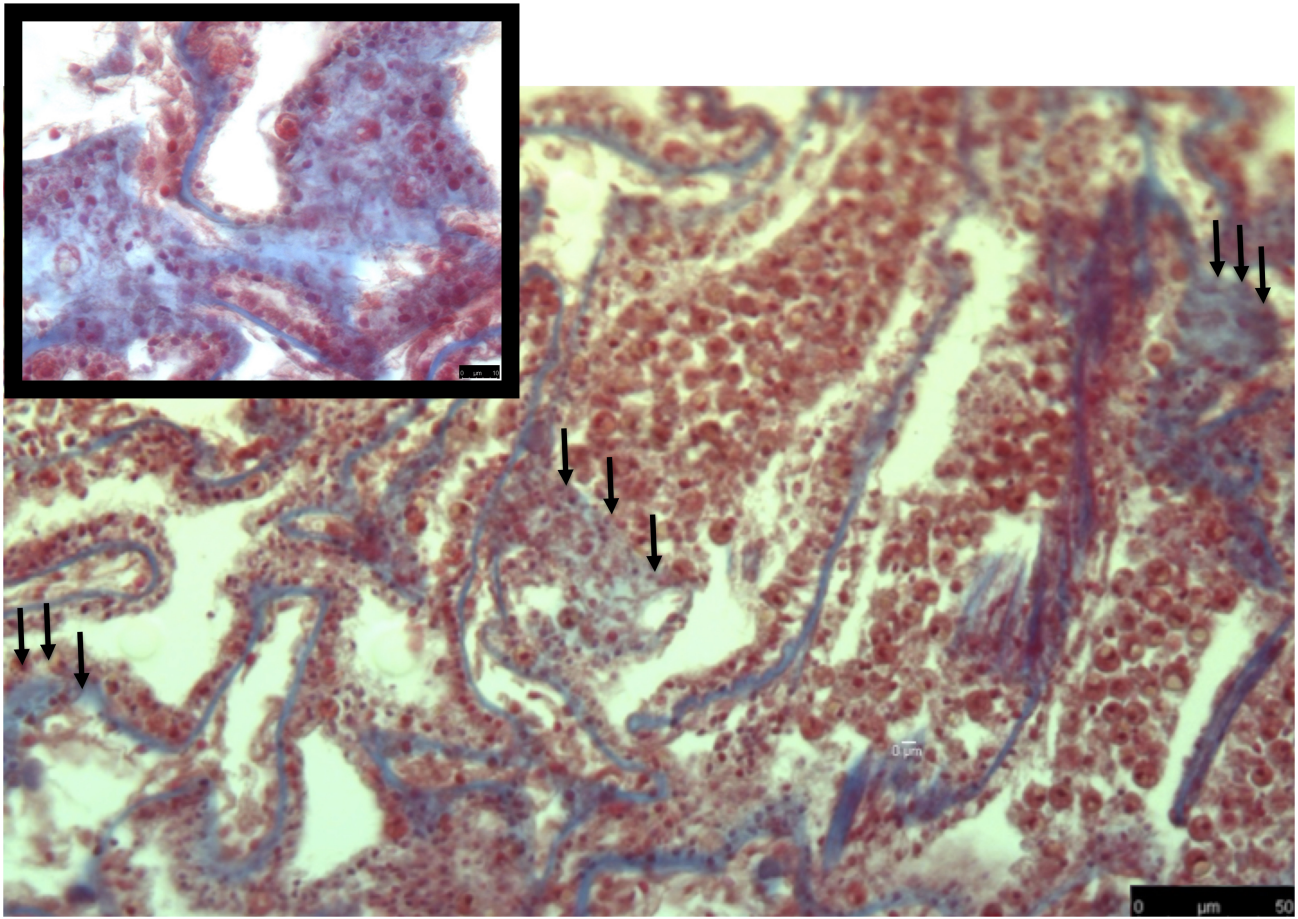
Masson’s trichrome-stained sections for collagen expression in heat-stressed coral samples. Heat-stressed samples have more diffuse collagen present (blue stain) through the gastrodermis. *Symbiodiniaceae* are showing signs of necrosis or are not present. Tissue structure is more damaged than what was observed in the control. Black arrows denote areas where collagen staining is expanded through the tissue area.

In this study I found evidence for heat stress causing a cellular pathology of necrosis and degradation, and the initiation of wound healing factors including collagen production (Figs. 1B, 2 and 3). The samples examined in this study had an average visual bleaching score of 2.3 out of 5 (Seneca & Palumbi, 2015; Rose, Seneca & Palumbi, 2015), indicating that colonies were partially bleached (Fig. S1). Slides of heat stressed coral stained with H & E had degradation and atrophy of the gastrodermis and the epidermis, along with shrunk and/or necrotic Symbiodiniaceae. Overall cell staining was very light, and cell walls were disrupted, damaged, and misshapen (Fig. 1B). Heat stress causes Symbiodiniaceae to produce large amounts of reactive oxygen species (ROS), which can overwhelm the cellular environment, causing Symbiodiniaceae to be released or degraded by many different cellular mechanisms (Nielsen, Petrou & Gates, 2018). Previously, in the coral *Acropora aspera*, cellular aspects of bleaching including apoptosis were observed in corals exposed to heat stress early in the bleaching response, indicating that the coral host was reacting to the heat stress long before the bleaching event occurred (Ainsworth et al., 2008). These cellular mechanisms cause a breakdown of the gastrodermis, which can leave a coral more vulnerable to pathogen invasion (Mydlarz et al., 2008; Palmer, Bythell & Willis, 2010).

### Collagen as a measurement of damage after a heat stress event

The common theme between previous transcriptome and microarray studies on heat stress in corals is that collagen, no matter what type, is reacting to heat stress. However, in corals the spatial location of where collagen is expressed after heat stress is not well understood. To address this, I used Masson’s trichrome to stain for collagen in heat stressed and control coral samples. In the control samples, collagen was present as part of the mesoglea (Fig. 2). However, in the heat stressed samples, areas of the tissue beyond the mesoglea had collagens present, including the epithelia (Figs. 2 and 3). The gastrodermis, which houses the Symbiodiniaceae, had collagen staining present as well (Fig. 3).

Early production and deposition of collagen after an acute heat stress event may be an important survival trait for corals that are subjected to high temperatures. The broad staining of collagen fibers in heat stress samples indicates that tissue rearrangement was occurring in response to heat stress. This is particularly surprising given that in the previous transcriptomic study on these same coral genotypes, collagen was down regulated in response to the same heat stress protocol (Seneca & Palumbi, 2015). This difference may be due to the type of collagen that was transcriptionally active in (Seneca & Palumbi, 2015), as well as, the types of collagens that are stained with Masson’s trichrome. This evidence indicates that the cellular damage caused by heat stress happens very quickly and early. Based on this observation, I hypothesize that collagen deposition may be an important mechanism that protects the coral in high temperature. In the future, it will be important to measure the rate and abundance of collagen that is deposited when a coral is recovering from a heat stress event, as this process could be an important recovery mechanism.

### Cnidarian grainyhead is found in three distinct clades, and is expressed in the gastrodermis of heat-stressed corals

Of the invertebrates that have previously been investigated only cnidarians have been found to have more than one GRH present in their genome (Traylor-Knowles et al., 2010). With the exception of *Hydra*, there has been an expansion of the GRH proteins within cnidarian linages, where 2–4 different paralogs of this protein are found (Fig. 3). The functional significance of this diversification is not understood, but it is possible that the function of this protein family is beyond wound healing. In mammals, *GRH* possesses many alternative splice sites, which enables *GRH* to produce many different types of protein products, thus increasing its overall ability to affect downstream wound healing genes (Miles, Dworkin & Darido, 2017). In this study we show that anthozoans have several different paralogs of *GRH,* which could possess many different alternative splice sites, potentially expanding their functional roles. Future studies on the alternative splices sites within cnidarian *GRH* may present interesting insights into the wide breadth of possible functional roles within cnidarians.

**Figure 4:**
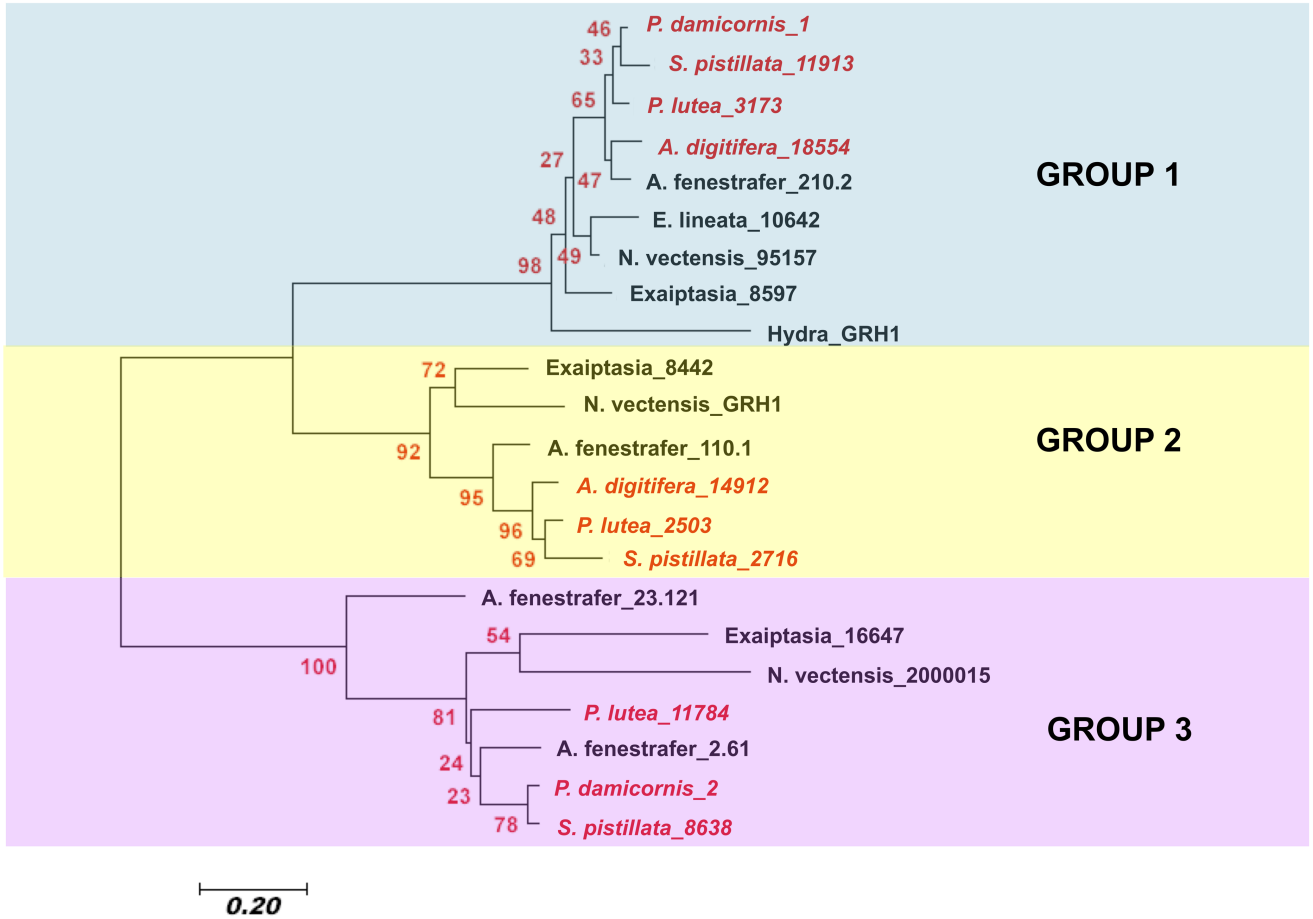
Neighbor-Joining tree of the evolutionary relatedness of Grainyhead proteins in cnidarians. The evolutionary history was inferred using the Neighbor-Joining method ( Saitou & Nei, 1987). The optimal tree with the sum of branch length = 5.13670293 is shown. The percentage of replicate trees in which the associated taxa clustered together in the bootstrap test (2,000 replicates) is shown next to the branches (Felsenstein, 1985). The tree is drawn to scale, with branch lengths in the same units as those of the evolutionary distances used to infer the phylogenetic tree. The evolutionary distances were computed using the Poisson correction method (Zuckerkandl & Pauling, 1965) and are in the units of the number of amino acid substitutions per site. The analysis involved 22 amino acid sequences. All positions with less than 95% site coverage were eliminated. That is, fewer than 5% alignment gaps, missing data, and ambiguous bases were allowed at any position. There were a total of 44 positions in the final dataset. Evolutionary analyses were conducted in MEGA7 (Kumar, Stecher & Tamura, 2016). Scleractinia is in red italic writing, while all other cnidarians are in black bold face. Sequences used in this study are found in Table S2.

To determine the evolutionary relationship of different cnidarian GRH proteins, a Neighbor-Joining phylogenetic analysis was used. The analysis involved 22 amino acid sequences and a total of 44 amino acid positions were analyzed in the final dataset (Fig. 4). I found that the different cnidarian GRH paralogs cluster into three different clades. Within clade 1, the GRHs concentrated into subclades according to taxa. Scleractinia is in one subclade, Actinaria in another, and *Hydra* was the most derived. Within clade 2 a similar pattern was observed, where Scleractinia and Actinaria formed separate subclades. Within group 3, resolution of Scleractinia and Actinaria was not as clear, with the protein sequence GRH A. fenenstrafer_23.121, being the most derived within the subclade. With the exception of group 3, the placement of the different GRH proteins indicates that the sequence evolution of this protein likely occurred within each taxonomic group, and may coincide with the diversification of specific traits of that taxonomic group.

Due to GRH’s conserved evolutionary role in maintaining the integrity of the epithelium and that several paralogs for this gene are found in corals, I next examined whether this gene was expressed in heat stressed coral tissue. *GRH* is a master transcription factor for wound healing and targets the activation of specific genes important to reestablishing the epithelium after a wound event (Mace, Pearson & McGinnis, 2005; Harden, 2005; Ting et al., 2005; Traylor-Knowles et al., 2010; Wang & Samakovlis, 2012). During wound healing, *GRH* is expressed in surface-lining epithelia of *Drosophila* and mice, and the cuticle secreted by the epithelium in *Drosophila* (Mace, Pearson & McGinnis, 2005; Ting et al., 2005). In cnidarians, little is understood about the role of *GRH* in epithelial integrity, but it is hypothesized that its role could be similar to what has been documented in *Drosophila* and mice wound healing (Mace, Pearson & McGinnis, 2005; Harden, 2005; Ting et al., 2005).

**Figure 5:**
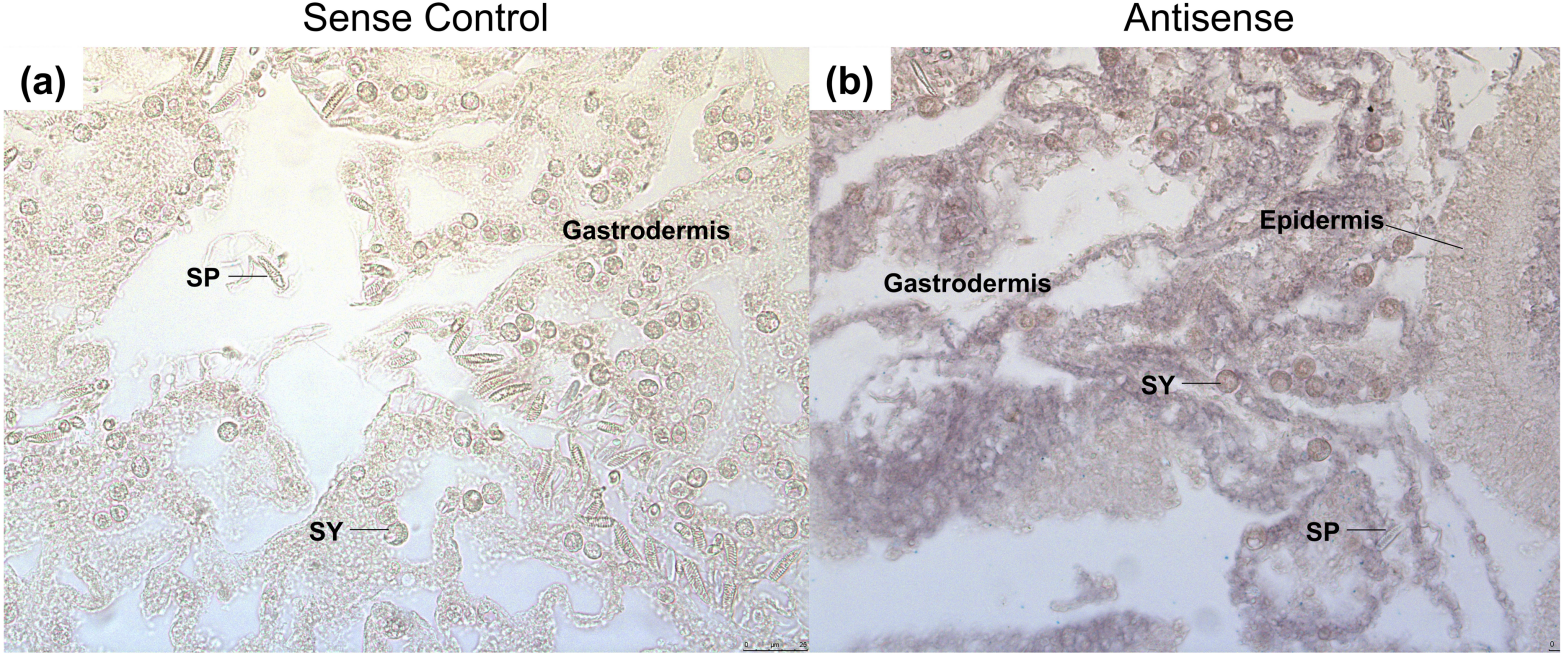
Grainyhead spatial expression in response to heat stress in corals. The following samples were done on serial sections from the same coral colonies. (A) The GRH sense control probe had no staining present. (B) Staining for the *GRH* anti-sense probe was found throughout the mesoglea and gastrodermis. Expression was not present within the spirocytes, but was found surrounding the *Symbiodiniaceae*, and the extracellular matrix of the gastrodermis layer. Black arrows point to purple staining of GRH antisense probe. Abbreviations: SY, *Symbiodiniaceae*; SP, Spirocyte

After heat stress, *GRH* is expressed throughout the gastrodermis of the coral (Figs. 5A and 5B). The expression is specific to the mesoglea adjacent to the gastrodermal cells, as well as, the cytoplasm of gastrodermal cells, which surround the Symbiodiniaceae (Fig. 5B). Staining was not found in the epidermis or within the nematocytes and spirocytes. In other organisms, the expression of *GRH* during wound healing is generally in the epidermis (Harden, 2005; Mace, Pearson & McGinnis, 2005; Tao et al., 2005; Ting et al., 2005; Wang & Samakovlis, 2012). This difference in expression may be due to the type of tissue damage, as well as the complexity of the tissue. For example, most studies on wound healing in *Dropsophila* and mice have been on subcutaneous, mechanical wounds, rather than wounds that were caused by heat (Harden, 2005; Mace, Pearson & McGinnis, 2005; Tao et al., 2005; Ting et al., 2005; Wang & Samakovlis, 2012).

## Conclusions

In this study, I show that heat stress has a physical effect on the tissue and the cells of the coral *A. hyacinthus,* possibly triggering wound-healing factors similar to those in other organisms. I demonstrated the physical effects by using histology to show that tissue integrity is compromised and that collagen is expressed throughout the gastrodermis of heat stressed corals. Additionally, these experiments revealed that *GRH*, a known transcription factor for epithelial integrity in other model organisms, was expressed throughout the gastrodermis of heat stressed corals. In fact, cnidarians possess three distinct groups of GRH proteins, suggesting the role of GRH in corals is potentially significant and worthy of further study. Significant progress has been made in understanding the disruptive effects of bleaching on coral tissue. Increasing our understanding of the heat stress recovery process of coral tissue allows us to identify corals with the potential to be more resilient. This knowledge will help us support the coral reef ecosystem, an environment that is crucially important for facilitating biodiversity, ocean health, and human health.

##  Supplemental Information

10.7717/peerj.6510/supp-1 Table S1 Coral colonies used for this experiment

10.7717/peerj.6510/supp-2 Table S2 Grainyhead sequence information for phylogenetics

10.7717/peerj.6510/supp-3 Table S3 Probe construction used for *in situ* hybridization

10.7717/peerj.6510/supp-4 Figure S1 Average visual bleaching score of heat stressed corals and control corals

## List of symbols and abbreviations

SY: Symbiodiniaceae
SU: symbiont containing gastrodermal cell
MU: mucocytes
NE: nematocytes
GRH: grainyhead
LB: lysogeny broth
PBS: phosphate buffered saline
PCR: polymerase chain reaction
SOC: Super Optimal broth with Catabolite repression
SSC: saline sodium citrate solution
ECM: extracellular matrix
LSF: Late SV40 Factor

## Additional Information and Declarations

### Data Availability

The following information was supplied regarding data availability: RNA probe and sequence acquisition information can be found in Tables S1–S3.
