# Supplementary figures and images for: Heat stress compromises epithelial integrity in the coral, Acropora hyacinthus
Source: PeerJ. 2019 Feb 26;7:e6510. doi: 10.7717/peerj.6510 (PMC6396749; doi:10.7717/peerj.6510)

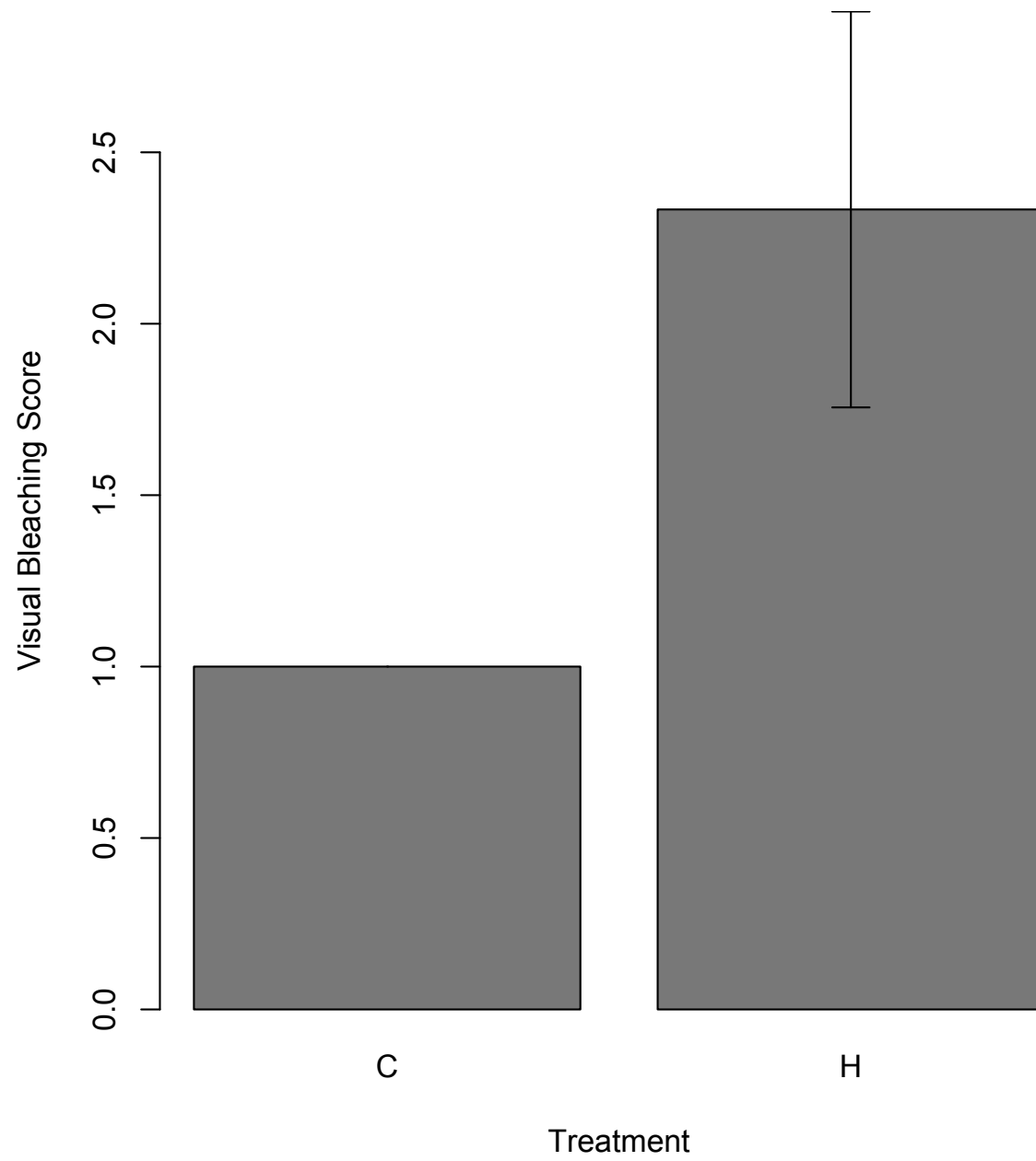

Supplement: Figure S1 [file peerj-07-6510-s004.pdf]
